# A retrospective comparison of respiratory events with JAK inhibitors or rituximab for rheumatoid arthritis in patients with pulmonary disease

**DOI:** 10.1007/s00296-021-04835-1

**Published:** 2021-03-15

**Authors:** Owen Cronin, Olivia McKnight, Lindsay Keir, Stuart H. Ralston, Nikhil Hirani, Helen Harris

**Affiliations:** 1 Rheumatic Diseases Unit, Western General Hospital, Crewe Road South, Edinburgh, EH4 2XU UK; 2 College of Medicine and Veterinary Medicine, University of Edinburgh, Edinburgh, UK; 3 Centre for Genomics and Experimental Medicine, Institute of Genetics and Molecular Medicine, University of Edinburgh, Western General Hospital, Edinburgh, UK; 4 Centre for Inflammation Research, University of Edinburgh, Edinburgh, UK; 5 Department of Respiratory Medicine, Royal Infirmary of Edinburgh, Edinburgh, UK

**Keywords:** Rheumatoid arthritis, Interstitial lung disease, Bronchiectasis, Janus kinases, Rituximab

## Abstract

Janus kinase inhibitors (JAKi) are an exciting option for the treatment of rheumatoid arthritis (RA) but little is known about their safety and tolerability in patients with existing respiratory disorders. The objective was to compare pulmonary safety of JAKi versus rituximab in patients with concurrent interstitial lung disease (ILD) or bronchiectasis. We performed a retrospective electronic patient record review of patients with known ILD or bronchiectasis commencing JAKi or rituximab for the treatment of RA. Patients initiating treatment from January 2016 to February 2020 were included. Respiratory events (hospitalization or death from a respiratory cause) were compared using Kaplan–Meier survival analysis. We analysed patients who received JAKi (*n* = 28) and rituximab (*n* = 19) for a mean (SD) of 1.1 (0.62) and 2.14 (1) years respectively. Patients were predominantly female (68%), anti-CCP antibody positive (94%) and non-smoking (89%) with a median (IQR) percentage predicted FVC at baseline of 100% (82–115%) and percentage predicted TL_CO_ of 62% (54.5–68%). Respiratory events occurred in five patients treated with JAKi (18%; 5 hospitalizations, 2 deaths) and in four patients treated with rituximab (21%; 3 hospitalizations, 1 death). Respiratory event rates did not differ between groups (Cox-regression proportional hazard ratio = 1.38, 95% CI 0.36–5.28; *p* = *0.64*). In this retrospective study, JAKi for the treatment of RA with existing ILD or bronchiectasis did not increase the rate of hospitalization or death due to respiratory causes compared to those treated with rituximab. JAK inhibition may provide a relatively safe option for RA in such patients.

## Introduction

Therapeutic decision-making in the treatment of rheumatoid arthritis (RA) for patients with concurrent pulmonary disease can be challenging. Fear regarding increased infection risk in bronchiectasis and progression of interstitial lung disease (ILD) secondary to drug administration restricts treatment options. Controversy exists as to whether medications, such as methotrexate, leflunomide and tumour necrosis factor-α inhibitors (TNFi), exacerbate pulmonary disease in patients with concurrent ILD and bronchiectasis [1]. This has led to hesitancy in utilizing these medications although systematic review of the evidence is beginning to allay fears [2, 3]. Recently, data have begun to support the safe and effective use of B and T cell therapies, rituximab [4] and abatacept [5], in the management of rheumatoid arthritis-related ILD and bronchiectasis [6, 7]. However, therapeutic options remain limited.

The approval of Janus kinase inhibitors (JAKi) (i.e., baricitinib, tofacitinib, upadacitinib) in the management of moderate to severe RA offers a new and exciting treatment paradigm [8–10]. However, inclusion of patients with concurrent ILD or bronchiectasis in relevant clinical trials is extremely limited. While the use of JAKi for the treatment of RA with co-existent lung disease is tempting due to the oral route of administration and relatively short half-life, their safety in relation to pulmonary toxicity in patients with established ILD or bronchiectasis is unknown.

To investigate this evidence gap, the objective of the current study is to compare the pulmonary safety of Janus kinase inhibition for the treatment of rheumatoid arthritis in patients with existing ILD or bronchiectasis, with rituximab. The primary outcome of interest was the incidence of severe respiratory events (hospitalization or death due to respiratory cause) in those receiving JAKi versus those receiving rituximab.

## Materials and methods

### Study design and patient inclusion

**Fig. 1:**
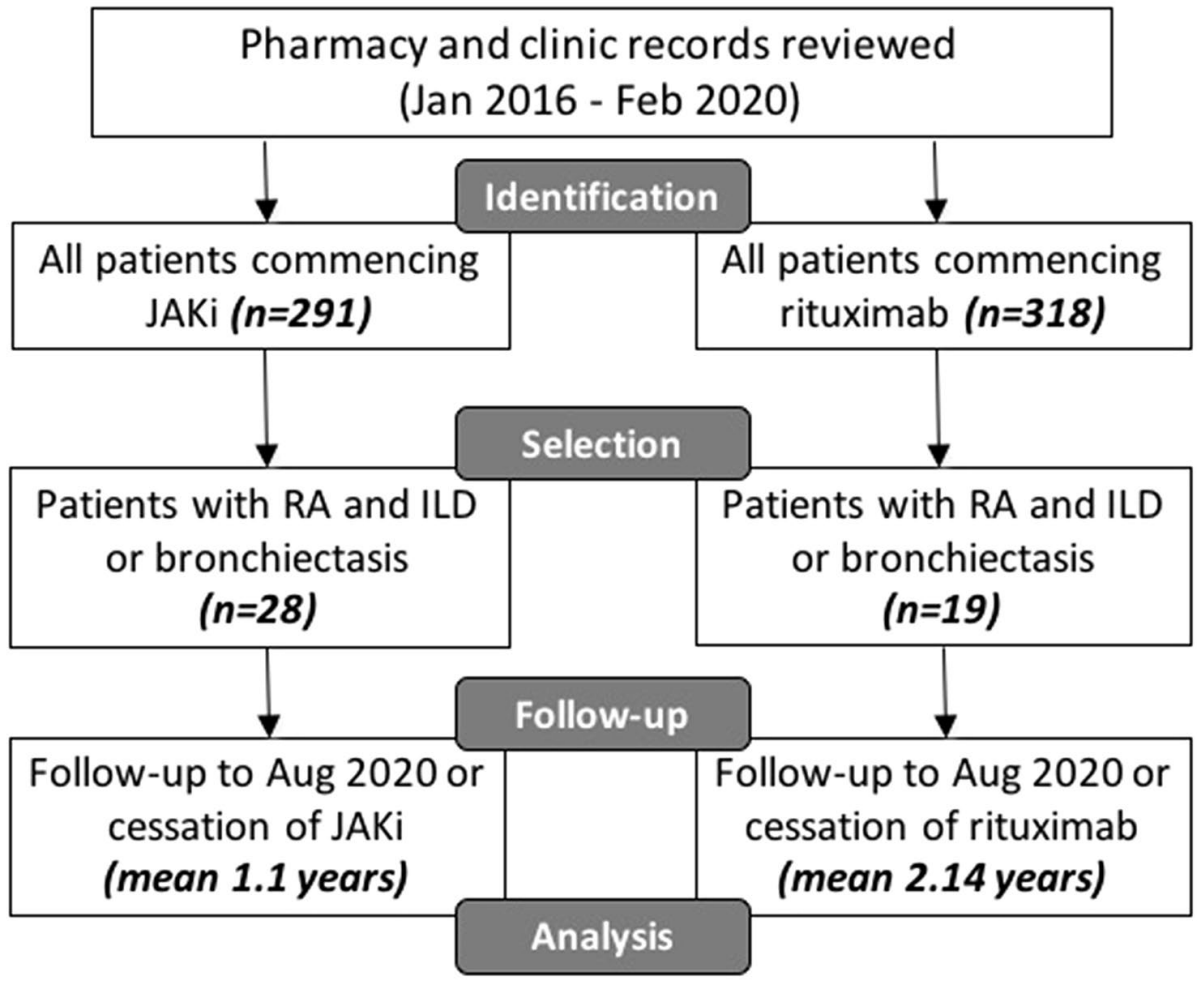
Study outline, selection of patients and follow-up

The study comprised a retrospective observational study. The electronic patient health record system (TrakCare®) was used to collect relevant data. Adult patients (aged 18 or over) undergoing treatment for rheumatoid arthritis at the Rheumatic Diseases Unit, Western General Hospital, Edinburgh, U.K., were included in the study. Only patients with an established diagnosis of ILD or bronchiectasis before the commencement of the drug of interest were eligible for inclusion. Patients without these respiratory conditions were excluded. Dedicated pharmacy and biologic clinic records were used to identify all adult patients (16 years or older) commencing any JAKi or rituximab for the treatment of RA from January 2016 to February 2020 (Fig. 1). From these records, all patients’ health records were screened by the investigators (OC, LK) for co-existing diagnoses of interstitial lung disease and/or bronchiectasis. For relevant patients, data were collected in detail from electronic health records by the investigators (OC, OM). Data were collected until discontinuation of the drug of interest (as documented in the clinical record) or to the end of the follow-up period (1 August 2020). They study was conducted in accordance with the Declaration of Helsinki. As all data were collected as part of routine clinical care and all treatment decisions were made prior to conduction of the study, ethical approval was not required.

### Clinical features and demographics

Identical clinical and demographic data were collected for both treatment groups. Standard clinical variables, such as age, gender, disease duration (rheumatoid arthritis and respiratory diseases), smoking history, past and current medications, anti-CCP antibody and rheumatoid factor (IgM) positivity, were collected along with the dates of commencement and cessation for the drugs of interest. Rheumatoid arthritis disease activity (DAS28-ESR or DAS28-CRP) before the commencement of the treatment of interest was recorded along with activity scores at 3–4 months post initiation (where available).

Baseline pulmonary function tests prior to the commencement of the medications of interest were also assessed. As only a small proportion of patients had follow-up pulmonary function assessment within a reasonable time frame, this was not analysed. The pattern of ILD involvement was described as recommended by the American Thoracic Society/European Respiratory Society following review of high-resolution (HR) CT-thorax imaging by a multi-disciplinary team including chest radiologists and an expert ILD lead physician (NH) who additionally attributed a modified semi-quantitative CT-fibrosis score by visual inspection [11, 12].

### Outcome measures

The primary outcome of interest was time to first respiratory event (days). Respiratory events were defined as either admission to hospital with a respiratory illness (e.g., infection, ILD exacerbation), or death from a respiratory cause while taking the medication of interest.

Additional outcomes of interest included drug continuation. Drug continuation was defined for JAKi as from the date of first prescription to the date of drug cessation, death, end of follow-up period, or whichever end-point came first. Drug continuation for rituximab was defined as from the date of first infusion to the date of cessation, death, end of follow-up period or whichever end-point came first. The number of cycles, frequency and dose of rituximab received was recorded for each patient.

### Statistical analysis

Statistical analysis was performed using IBM SPSS statistics v.25.0 (IBM, Armonk, New York, USA). Distributions of clinical variables and demographics were illustrated using histograms. Depending on the data distribution and type (i.e., categorical, continuous), variables were then described using means (with standard deviations) or medians (with inter-quartile ranges). Continuous variables were compared for differences between groups using either student’s *t* tests or Mann–Whitney *U* testing subject to normality or non-normality of distribution. Proportions of categorical variables were compared using Chi-squared testing. Kaplan–Meier survival analysis (log-rank) was used to compare respiratory event survival and drug continuation survival between treatment groups. Cox regression proportional hazard was used to calculate unadjusted hazard ratios with 95% confidence intervals (CI 95%). Statistical significance was set at *p* < *0.05* and analysis was two -tailed.

## Results

### Demographic and clinical characteristics

A total of 291 patients were commenced on JAKi to February 2020 at the Rheumatic Diseases Unit, Edinburgh (Fig. 1). Twenty-eight patients commenced JAK inhibition for the treatment of rheumatoid arthritis who also had concurrent interstitial lung disease (67.9%), bronchiectasis (25%) or both (7.1%). Baricitinib was commenced in 26 patients and tofacitinib in 2 patients. A total of 318 patients were commenced on rituximab from January 2016 to February 2020 (Fig. 1). Nineteen patients commenced rituximab for the treatment of RA and had concurrent ILD (68.4%), bronchiectasis (26.3%) or both (5.3%).

Mean duration of follow-up (to discontinuation of drug, death or end of follow-up period) for patients receiving JAKi was 1.1 years (SD = 0.62) and for patients receiving rituximab infusions was 2.14 years (SD = 1). Demographic and clinical characteristics were similar in both treatment groups (Table 1); however, those commencing JAKi had a significantly longer disease duration (median 11 years) compared to those starting rituximab (median 3 years) (Table 1; Mann–Whitney *U* test, *p* = *0.003*).

**Table 1:** Demographic and clinical details of included patients

	JAKi (*n* = 28)	Rituximab (*n* = 19)	No. of patients with available data (*n* =)
ILD/Bronchiectasis/both *n, (%)*	ILD *n* = 19 (67.9%) Bronchiectasis *n* = 7 (25%) Both *n* = 2 (7.1%)	ILD *n* = 13 (68.4%) Bronchiectasis *n* = 5 (26.3%) Both *n* = 1 (5.3%)	47
Age *(median, IQR)*	69 (62.3–75)	70 (59–76)	47
Female *n, *(%)	18 (64.3%)	14 (73.7%)	47
Smoking status *n, *(%)	Smoker: *n* = 5 (17.9%) Ex-smoker: *n* = 15 (53.6%) Never-smoker: *n* = 8 (28.6%)	Smoker: *n* = 0 (0%), Ex-smoker: *n* = 12 (63.2%) Never-smoker: *n* = 7 (36.8%)	47
Anti-CCP positivity *n, (%)*	25 (96.2%)	19 (100%)	45
Rh-Factor IgM positivity *n, (%)*	14 (77.8%)	9 (90%)	28
RA diagnosis age *median, (IQR)*	53 (44–61)	54 (48–67)	42
Respiratory diagnosis age *median, (IQR)*	64 (55–73)	64 (53–71)	46
Years from RA diagnosis to drug of interest *median, (IQR)*	11 (3–20)	3 (1–6)	42
DAS-28 _Baseline_ *median, (IQR)*	5.85 (4.98–6.18)	6.2 (5.53–6.86)	43
DAS-28 _3–4 months_ *median, (IQR)*	3.4 (2.38–4.11)	4.2 (3.61–5.44)	32

*JAKi* Janus kinase inhibitor, *ILD* interstitial lung disease, *Rh-factor* rheumatoid factor, *RA* rheumatoid arthritis, *DAS-28* disease activity score 28 joints

Pre-treatment pulmonary function tests indicated mild severity of pulmonary disease in patients commencing JAKi and rituximab (Table 2). Review of patients’ baseline high-resolution CT-thorax revealed similar radiological patterns and severity of ILD in both treatment groups (Table 2). However, a greater degree of ground glass was evident on CT-imaging before treatment in those receiving JAKi compared to those receiving rituximab (*p* = *0.036*).

**Table 2:** Baseline pulmonary function (all patients); and radiological pattern and severity of ILD on CT-thorax imaging (patients with ILD)

	JAKi (*n* = 28)	Rituximab (*n* = 19)	No. of patients with available data (*n* =)
Baseline predicted FEV_1_ *median %, (IQR)*	93.5 (74.5–105)	92 (76–105)	43
Baseline predicted FVC *median %, (IQR)*	103 (78–112)	99 (86–119)	44
Baseline predicted TL_CO_ *median %, (IQR)*	62 (51.25–69)	62 (55–67)	41

Radiological ILD-pattern	JAKi (*n* = 20)	Rituximab (*n* = 14)	
UIP *n, *(%)	0 (0%)	2 (14%)	
Probable UIP *n, *(%)	4 (20%)	5 (36%)	
Fibrotic NSIP *n, (%)*	6 (30%)	1 (7%)	
Chronic HP *n, *(%)	3 (15%)	1 (7%)	
Respiratory bronchiolitis-ILD *n, *(%)	1 (5%)	0 (0%)	
Unclassifiable *n, *(%)	6 (30%)	5 (36%)	
% of lung with ILD *median, (IQR)*	20 (10–45)	12.5 (10–30)	
% of lung with ground glass appearance *median, (IQR)*	20 (5–45)	7.5 (5–10)	
% of lung with honeycombing *median, (IQR)*	0 (0–1.75)	0 (0–2)	
% of lung with emphysema *median, (IQR)*	0.5 (0–8.75)	0 (0–3.75)	

*JAKi* Janus kinase inhibitor, *ILD* interstitial lung disease, *FEV*_*1*_ forced expiratory volume in 1 second, *FVC* forced vital capacity, *TL*_*co*_ lung transfer capacity for carbon monoxide, *IQR* inter-quartile range, *UIP* usual interstitial pneumonia, *NSIP* non-specific interstitial pneumonia, *HP* hypersensitivity pneumonitis

### Treatment details

Concurrent treatments including conventional synthetic DMARDs and corticosteroids were similar in both groups (Table 3). Almost 30% of patients were biologic naïve before commencing the drug of interest. Prior treatment history indicated that those commencing JAKi had previously been treated with a greater number of biologics including TNFi, tocilizumab and abatacept, compared to those commencing rituximab.

**Table 3:** Previous and concurrent DMARD treatments

	JAKi (*n* = 28)	Rituximab (*n* = 19)
Previous treatments		
No. of previous csDMARDs *median, (IQR)*	3 (2–4)	3 (2–4)
Methotrexate *n, *(%)	22 (78.6%)	13 (68.4%)
Biologic Naïve *n, *(%)	8 (28.6%)	6 (31.6%)
No. of previous biologics *median, (IQR)*	1 (0–2)	0 (0–1)
Rituximab/JAKi *n, *(%)	12 (42.9%)	1 (5.3%)
TNF-α inhibitor *n, *(%)	15 (53.6%)	5 (26.3%)
Tocilizumab *n, *(%)	6 (21.4%)	1 (5.3%)
Abatacept *n, *(%)	6 (21.4%)	1 (5.3%)
Concurrent treatments		
Prednisolone; *n,* (%)	13 (46.4%)	6 (31.6%)
Prednisolone dose (mg) * median, (IQR)*	7 (5–10)	10 (4.5–12.5)
Methotrexate *n, *(%)	4 (14.3%)	2 (10.5%)
Azathioprine *n, *(%)	2 (7.1%)	2 (10.5%)
Leflunomide *n, *(%)	2 (7.1%)	2 (10.5%)
Hydroxychloroquine *n, *(%)	6 (21.4%)	6 (31.6%)
Sulfasalazine *n, *(%)	3 (10.7%)	4 (21.1%)

*DMARD* disease-modifying anti-rheumatic drugs, *csDMARDs* conventional synthetic DMARDs, *JAKi* Janus kinase inhibitor, *IQR* inter-quartile range, *TNF* Tumour necrosis factor

All except for two patients received standard licensed dosing regimens for JAK inhibition for the treatment of RA (i.e. baricitinib 4 mg once daily; tofacitinib 5 mg twice daily). One patient took 4 mg/2 mg of baricitinib on alternate days and one patient took 5 mg of tofacitinib once daily before increasing to 5 mg twice daily.

All patients who received rituximab received the standard dosing of treatment (2 × intravenous infusions of 1000 mg rituximab with 100 mg intravenous methylprednisolone 14 days apart). Only 2/19 patients were on ‘fixed’ cycles of rituximab therapy repeated every 6 months. All others received rituximab as required depending on clinical status but not within 6 months of the last infusion. 5/19 patients received only 1 cycle of rituximab during their study follow-up period. All others (74%) received between 2 and 5 cycles of rituximab.

### Incidence of respiratory events

**Fig. 2:**
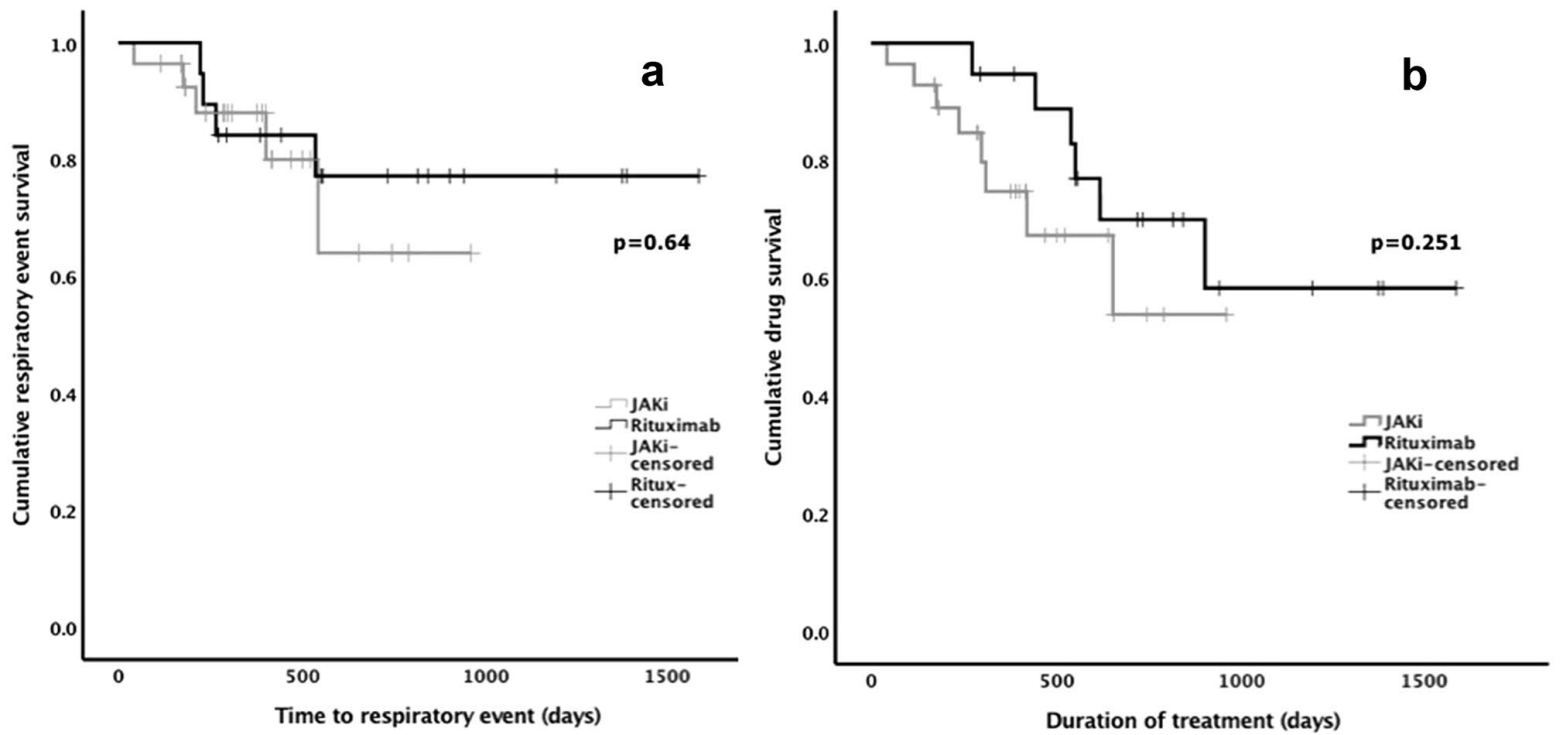
**a** Comparison of time to respiratory event and **b** drug continuation between JAKi and rituximab for the treatment of rheumatoid arthritis in patients with concurrent bronchiectasis and/or ILD

Respiratory events (i.e. hospitalization or death due to a respiratory cause) occurred in five patients (18%) during JAKi treatment (7 hospitalizations, two of which led to death) and in four patients (21%) (4 hospitalizations, one of which led to death) during treatment with rituximab. In the JAKi group, two patients (7.1%) died of respiratory issues (one new lung cancer with concomitant community acquired pneumonia and one of community acquired pneumonia). In the rituximab-treated patients, two patients (10.5%) died during follow-up; one of an acute myocardial infarction and one of pneumonitis and respiratory failure. No episodes of acute pneumonitis occurred in the JAKi group. There was no difference in respiratory event survival in the JAKi-treated group compared with the rituximab-treated group (Fig. 2a); unadjusted hazard ratio: 1.38 (95% CI 0.36, 5.28); *p* = *0.64*.

### Drug continuation

Seventy-one percent (20/28) of patients with ILD and/or bronchiectasis who commenced JAKi for the treatment of rheumatoid arthritis remained on the drug at the end of the follow-up period. Reasons for discontinuation included inefficacy (*n* = 3), death (*n* = 2, as outlined above), recurrent infections (*n* = 2) and reduced renal function (*n* = 1).

Sixty-three percent (12/19) of patients who commenced rituximab continued the drug to the end of the follow-up period. Reasons for cessation included inefficacy (*n* = 5) or death (*n* = 2, as outlined above). The rate of discontinuation of JAKi in patients with ILD and/or bronchiectasis did not significantly differ from that of those receiving rituximab (Fig. 2b); unadjusted hazard ratio: 1.9 (95% CI 0.634, 5.73); *p* = *0.251*.

## Discussion

There is an absence of patients with interstitial lung disease and bronchiectasis in randomised controlled trials that examine the effectiveness of JAK inhibitors for the treatment of rheumatoid arthritis. Therefore, we have limited knowledge of the pulmonary safety in these cohorts. Prevalence of interstitial lung disease and bronchiectasis is relatively high in patients with rheumatoid arthritis, estimated to affect between 5 and 10% of patients [1, 13]. Interstitial lung disease itself contributes significantly to the excess mortality evident in patients with rheumatoid arthritis [14]. Despite this, the optimal treatment strategy for such patients remains unknown, reflecting a paucity of randomized controlled clinical trials for this patient sub-group. Recently, observational data have suggested treatment with rituximab [6, 15, 16], and laterally abatacept [5, 17] is the most effective and safest biologic agents for the treatment of RA with concurrent ILD or bronchiectasis. Data suggest that treatment with rituximab and abatacept may even infer a benefit to pulmonary function [5, 6]. Nonetheless, concern about lung toxicity restricts the use of many DMARDs and biologic agents, be these concerns soundly proven or not.

Novel, small-molecule treatments, such as Janus kinase inhibitors, represent an exciting option for patients with rheumatoid arthritis. Their safety and potential for effectiveness in patients with concurrent pulmonary disease should be duly considered. Currently, our understanding of inhibition of JAK/STAT signalling pathways in patients with ILD and bronchiectasis is extremely limited. JAKi-use has led to successful treatment of dermatomyositis-associated ILD but evidence is restricted to a small number of case reports and case series [18–20]. Even less is understood about Janus kinase inhibition in patients with bronchiectasis. While post hoc analysis of phase 2, 3 and 3b/4 clinical trials indicates that the de novo incidence of interstitial lung disease is low in patients with rheumatoid arthritis treated with JAKi [21]; to our knowledge, no study has yet examined the pulmonary tolerance and safety of JAKi in those with existing ILD or bronchiectasis. The current study sought to address this important clinical scenario, by comparing time to respiratory events in patients receiving JAKi with patients receiving the current, best-practice medication for this clinical cohort, rituximab.

The majority of our patients commenced JAK1/2 inhibition with baricitinib due to the earlier licensing of this medication for the treatment of rheumatoid arthritis but several patients commenced JAK1/3 inhibition with tofacitinib. Furthermore, the proportion of patients with ‘difficult-to-treat’ rheumatoid disease, manifest by a longer duration of illness and a greater number of previous biologic therapies (e.g. tocilizumab, abatacept), was higher in those subsequently prescribed JAKi. We believe this relates to the fact that a novel medication provides an attractive option for patients who have had difficult-to-treat disease for many years and in such cases patients often chose to switch therapy in an attempt to achieve remission.

Encouragingly, serious respiratory events (i.e., hospitalization or death due to a respiratory cause) were no different between those treated with JAKi and those treated with rituximab suggesting that JAKi may offer a realistic treatment option for patients with RA and concurrent ILD or bronchiectasis. Our cohort of patients represents a group with mild to moderate pulmonary disease, all of whom commenced Janus kinase inhibition or rituximab with a known, pre-existing diagnosis of ILD or bronchiectasis (present on cross sectional CT-imaging and/or attending a tertiary ILD clinic). Very few patients with ILD had a HR-CT pattern consistent with definite UIP and most had a pattern that was consistent with an alternative diagnosis and were highly unlikely to be UIP. Therefore, our findings should not be extrapolated to those with severe ILD or bronchiectasis or a definite UIP pattern of disease and caution is urged in this setting. Furthermore, there was no significant difference in time to drug discontinuation between those prescribed JAKi and those prescribed rituximab. Proportionally, more patients treated with rituximab (almost 40%) ceased treatment during follow-up compared with those treated with JAKi (almost 30%). Reasons for discontinuation of rituximab predominantly related to clinical inefficacy, but comparison of clinical effectiveness was not the aim of this study.

There are several strengths to this study. Both cohorts of patients (i.e., JAKi- and rituximab-receiving) were similar in terms of clinical traits (e.g. gender, concurrent medications, corticosteroid use, anti-CCP and rheumatoid factor seropositivity) minimizing the effect of confounders on respiratory event outcomes. In addition, these data represent real-world experience of patients with RA and concurrent ILD/bronchiectasis with patient–clinician decisions made without influence of a pre-determined research goal. The retrospective nature of this observational study and associated risk of information bias represents the major limitation of this study. The relatively small sample size and short follow-up period also represent limitations that may have affected overall results. Ideally, follow-up pulmonary function testing would have been available for all patients after commencing treatment but as this was only the case for a minority of patients, this was not analysed. It was not possible to collect primary care records of respiratory exacerbations or infections that did not require hospital admission that were treated in the community. Furthermore, the electronic patient health record system used did not permit recording of admissions to hospital abroad or to hospitals outside of NHS Lothian. The authors recommend that the findings of this study and analysis should be investigated further in larger prospective cohorts in the future.

To conclude, within the limitations of this retrospective analysis, JAK inhibition with baricitinib or tofacitinib for the treatment of rheumatoid arthritis in patients with concurrent interstitial lung disease or bronchiectasis did not increase the rate of hospitalization or death due to a respiratory cause in comparison to those treated with rituximab. JAK inhibition may provide a favourable and safe therapeutic option for such patients but larger, prospective studies are required to confirm these findings to inform much-needed therapeutic guidance in this sub-category of patients with RA and ILD/bronchiectasis.
